# A posteriori model validation for the temporal order of directed functional connectivity maps

**DOI:** 10.3389/fnins.2015.00304

**Published:** 2015-08-27

**Authors:** Adriene M. Beltz, Peter C. M. Molenaar

**Affiliations:** Department of Human Development and Family Studies, The Pennsylvania State UniversityUniversity Park, PA, USA

**Keywords:** a posteriori model validation, directed functional connectivity, neuroimaging, structural vector autoregression, temporal order, unified structural equation modeling

## Abstract

A posteriori model validation for the temporal order of neural directed functional connectivity maps is rare. This is striking because models that require sequential independence among residuals are regularly implemented. The aim of the current study was (a) to apply to directed functional connectivity maps of functional magnetic resonance imaging data an a posteriori model validation procedure (i.e., white noise tests of one-step-ahead prediction errors combined with decision criteria for revising the maps based upon Lagrange Multiplier tests), and (b) to demonstrate how the procedure applies to single-subject simulated, single-subject task-related, and multi-subject resting state data. Directed functional connectivity was determined by the unified structural equation model family of approaches in order to map contemporaneous and first order lagged connections among brain regions at the group- and individual-levels while incorporating external input, then white noise tests were run. Findings revealed that the validation procedure successfully detected unmodeled sequential dependencies among residuals and recovered higher order (greater than one) simulated connections, and that the procedure can accommodate task-related input. Findings also revealed that lags greater than one were present in resting state data: With a group-level network that contained only contemporaneous and first order connections, 44% of subjects required second order, individual-level connections in order to obtain maps with white noise residuals. Results have broad methodological relevance (e.g., temporal validation is necessary after directed functional connectivity analyses because the presence of unmodeled higher order sequential dependencies may bias parameter estimates) and substantive implications (e.g., higher order lags may be common in resting state data).

## Introduction

A posteriori validation of models used for *directed* functional connectivity mapping is rare in neuroscience. In directed functional connectivity maps, neuroscientists use time-indexed data to model contemporaneous (e.g., with structural equation models, or SEMs; McIntosh and Gonzalez-Lima, 1994) and lagged (e.g., with vector autoregressive models, or VARS; Penny and Harrison, 2007) connections among brain regions (Friston et al., 2013), but they rarely check to see if the obtained results provide the best representation of the data. Model fit is a necessary, but not a sufficient, indicator: A model can fit the data well according to absolute and alternative fit indices, even when key features of the data are not modeled or when alternative models have an equivalent fit to the data (Lütkepohl, 2005; Ramsey et al., 2010; Beltz and Molenaar, under review). Thus, a posteriori model validation has implications for the accuracy of substantive inferences and for replicability, as convergent evidence is unlikely to emerge from results obtained with suboptimal modeling procedures (Pashler and Wagenmakers, 2012).

A key element of a posteriori model validation of directed functional connectivity maps concerns the temporal order of the model, focusing on the presence of unmodeled sequential dependencies. Were the modeled lags sufficient to remove all auto- and cross-correlations from the data, and thus, to create white noise residuals? Even though it is rarely implemented in neuroscience, a posteriori model validation for temporal orders is not new (see Box and Jenkins, 1970). The failure to address the impact of correlated residuals in econometric time series models that assume independent residuals was criticized in seminal work over 40 years ago (Granger and Newbold, 1974). The identification of the appropriate lag for a data set by fitting and comparing time series models of multiple temporal orders was also discussed 40 years ago in decisive mathematical statistics work (Akaike, 1974).

Today in neuroscience, sequential dependencies in a data set are usually handled prior to inferential statistical tests. For example, it is standard practice to remove noise (e.g., cardiac and respiratory signal) using autoregressive processes to whiten or bandpass filter functional magnetic resonance imaging (fMRI) data in general linear model (GLM) analyses in order to ensure that residuals are white noise and without bias (e.g., Friston et al., 2000; Woolrich et al., 2001; Penny et al., 2003). Moreover, there have been recent investigations (Christova et al., 2011; Kaneoke et al., 2012; Arbabshirani et al., 2014) into the role of auto- and cross-correlations in *undirected* functional connectivity maps (Friston et al., 2013). The focus of these investigations has been on fMRI resting state data because they are straight-forward (i.e., there is no need to model different patterns of autocorrelations across task conditions) and because typical analysis pipelines for resting state data correlate the raw time series from all voxels or several brain regions of interest (ROIs), assuming that the modeled contemporaneous connections (i.e., relations with zero order lags, such as Pearson correlations) are not confounded by unmodeled sequential dependencies. These analysis pipelines are biased, however, as auto- and cross-correlations have been shown to impact the validity of obtained maps, producing inaccurate parameter values and even spurious connections (Christova et al., 2011; Kaneoke et al., 2012; Arbabshirani et al., 2014). Nonetheless, it has been argued that corrections for autocorrelations do not affect substantive inferences drawn from hypothesis testing of undirected functional connectivity map parameters (e.g., inferences about group differences), presumably because corrections have similar influences on means and standard deviations (Arbabshirani et al., 2014).

Despite the prevalence of accounting for sequential dependences in GLM analyses and recent interest in the role of auto- and cross-correlations in undirected functional connectivity mapping, issues surrounding model order selection in directed functional connectivity mapping continue to be underappreciated. In other words, it is uncommon for neuroscientists to establish that the maximum order of the lag in their directed functional connectivity maps is sufficient for capturing all sequential dependencies in their fMRI data; they do not demonstrate that the model residuals are white noise, even though this is an explicit assumption of the models (McIntosh and Gonzalez-Lima, 1994; Penny and Harrison, 2007). This assumption may often be violated because common methods for mapping directed functional connectivity only concern contemporaneous *or* lagged connections among ROIs, and thus, fail to account for various types of temporal processes within the same model. For example, path models, such as SEMs, only model contemporaneous connections among ROIs. A SEM with a mean fixed at zero is defined as:

(1) y(t)=Δy(t) + ξ(t),

where y(t) is the p-variate time series to be explained at repetition time (TR) *t* = 1, 2,…, T, with T the length of the time series; Δ is the (p,p)-dimensional matrix of contemporaneous regression coefficients, and ξ is the p-variate process innovations, theoretically lacking sequential dependencies and having a zero mean and a diagonal contemporaneous covariance matrix. Furthermore, VARs only model lagged connections among ROIs. A standard VAR(a) with a mean fixed at zero is defined as:

(2) $$\text{y}(\text{t})=\Theta_{1}\text{y}(\text{t}-1)\;+\;\Theta_{2}\text{y}(\text{t}-2)\;+\;\ldots \;+\;\Theta_{\text{a}}\text{y}(\text{t}-\text{a})\;+\text{ ε}(\text{t}),$$

where y(t) is defined as in Equation (1); Θ_*q*_ is the (p,p)-dimensional matrices of regression coefficients at lag *q* = 1, 2,…, a, and ε is the p-variate process innovations, theoretically lacking sequential dependencies and having a zero mean and full covariance matrix.

Structural VARs, however, consider both contemporaneous and lagged directed functional connections among ROIs, and thus, incorporate more information about temporal processes than do methods for undirected functional connectivity mapping (e.g., Pearson correlations) and alternative models for directed functional connectivity mapping, such as SEMs and VARs. A structural VAR(a) with a mean fixed at zero is defined as:

(3) $$\begin{array}{l} \text{y}(\text{t})=\Gamma \text{y}(\text{t})\;+\;\Lambda_{1}\text{y}(\text{t}-1)\;+\;\Lambda_{2}\text{y}(\text{t}-2)\;+\;\ldots \;\\ \;+\;\Lambda_{\text{a}}\text{y}(\text{t}-\text{a})\;+\text{ ν}(\text{t}), \end{array}$$

where y(t) is defined as in Equation (1), Γ is the (p,p)-dimensional matrix of contemporaneous regression coefficients, Λ_q_ is the (p,p)-dimensional matrices of regression coefficients at lag *q* = 1, 2,…, a, and ν is the p-variate process innovations, theoretically lacking sequential dependencies and having a zero mean and a diagonal contemporaneous covariance matrix. There are several types structural VARs (e.g., Kim et al., 2007; Chen et al., 2011), differing primarily in the statistical procedures through which the contemporaneous connections are obtained. The most common way to obtain structural VARs is to transform the standard VAR in Equation (2) by means of Cholesky decomposition of the full covariance matrix of the process innovations ε(t) (Lütkepohl, 2005). But, this approach is problematic, as the contemporaneous connections are dependent upon the ordering of the univariate component series in y(t) (see Loehlin, 1996; Lütkepohl, 2005; Molenaar and Lo, 2015).

### The unified structural equation model (uSEM) family of approaches

Another way to obtain structural VARs—and to avoid dependencies based on the ordering of the input series—is by using the unified SEM (uSEM; Gates et al., 2010). A uSEM(a) with a mean fixed at zero is defined as:

(4) $$\begin{array}{l} {\text{η}}_{i}(\text{t})={\text{Aη}}_{i}(\text{t})\;+\;\Phi_{1}{\text{η}}_{i}(\text{t}-1)\;+\;\Phi_{2}{\text{η}}_{i}(t-2)\;+\;\ldots \\ \;+\;\Phi_{\text{a}}{\text{η}}_{i}(\text{t}-\text{a})+{\text{ζ}}_{i}(\text{t}), \end{array}$$

where η_*i*_(t) is the p-variate time series to be explained for individual *i* at TR *t* = 1, 2,…, T, with T the length of the time series, A is the (p,p)-dimensional matrix of contemporaneous regression coefficients, Φ_q_ is the (p,p)-dimensional matrices of regression coefficients at lag *q* = 1, 2,…, a, and ζ_*i*_ is the p-variate process innovations, theoretically lacking sequential dependencies and having a zero mean and a diagonal contemporaneous covariance matrix.

The primary difference between common structural VARs and uSEMs is not in statistical formulation [e.g., notice that Equations (3) and (4) differ only in symbolic notation], but rather in the way in which the models are fit to the data. In order to provide accurate solutions (unlike the solutions of structural VARs fitted by means of standard VARs followed by Cholesky decomposition of the innovations that depend upon the ordering of the input series), uSEMs directly fit Equation (4) to the data using a special estimation procedure (described below and in Gates et al., 2010). This confers several advantages. First, uSEMs can be estimated in a confirmatory or data-driven manner. In a confirmatory application, the Equation (4) structures of A and Φ_q_ (i.e., the pattern of relations among ROIs to be estimated) are pre-specified. In a data-driven application, the structures of A and Φ_q_ are determined via sequential Lagrange Multiplier testing of modification indices (cf. Gates et al., 2010).

Second, the fitting of a data-driven uSEM is not affected by the ordering of the ROIs. A null model [i.e., Equation (4) with empty A and Φ_q_ matrices] is fit to the data, then the significant connection between two ROIs with the largest modification index (i.e., the relation that will most improve model fit; Sörbom, 1989) is freed, with the model iteratively re-estimating and freeing the connection with the highest modification index until the freeing of a connection will no longer significantly improve model fit. Sequential model fitting is then followed by the removal of non-significant parameters via the Wald test. This procedure has been shown to perform as well as the likelihood ratio test in covariance structure modeling (Chou and Bentler, 1990).

Third, the extended version of uSEM (euSEM; Gates et al., 2011) can incorporate external input, or vectors of task conditions. An euSEM(a) with a mean fixed at zero is defined as:

(5) $$\begin{array}{l} {\text{η}}_{i}\left(\text{t}\right)={\text{Aη}}_{i}\left(\text{t}\right)+\sum_{q\text{=1}}^{\text{a}}\Phi_{q}{\text{η}}_{i}\left(\text{t}-\text{ q}\right)\;+\;\sum_{r\text{=0}}^{\text{f}}\gamma_{r}{\text{u}}_{i}\left(\text{t}-\text{r}\right)\\ \;+\sum_{q\text{=1}}^{g}\sum_{r\text{=1}}^{h}\tau_{q,r}{\text{η}}_{i}\left(\text{t}-\text{q}\right){\text{u}}_{i}\left(\text{t}-\text{r}\right)\;+\;{\text{ζ}}_{i}(\text{t}) \end{array}$$

where η_*i*_(t), *i*, A, Φ, q, a, and ζ_*i*_ are defined as in Equation (4), γ is the (p,1)-dimensional matrices of regression coefficients for direct effects of the hemodynamic response function (HRF)-convolved external input vectors, u, on ROIs at lag *r* = 0, 1,…, f, and τ_*q, r*_ is the (p,p)-dimensional matrices of regression coefficients for the modulating effects of u on connections between ROIs at lags *q* = 1, 2,…, g and *r* = 1, 2,…, h. An euSEM is fit to the data in the same manner as a uSEM. A confirmatory or data-driven approach can be implemented, and for the latter, modification indices are used to iteratively free connections in A, Φ, γ, and τ until the freeing of a connection will no longer significantly improve model fit; the model is then trimmed of non-significant parameters.

Fourth, data-driven uSEMs can be implemented within a grouping algorithm in order to make inferences about heterogeneous, multi-subject data using group iterative multiple model estimation (GIMME; Gates and Molenaar, 2012). GIMME with a mean fixed at zero is defined as:

(6) $$\begin{array}{l} {\text{η}}_{i}\left(\text{t}\right)=({\text{A}}_{i}\;+\;{\text{A}}^{g}){\text{η}}_{i}\left(\text{t}\right)\text{ + }\sum_{q=1}^{a}(\Phi_{i,q}\;+\;\Phi_{q}^{g}){\text{η}}_{i}\left(\text{t}-q\right)\\ \text{ + }\sum_{r=0}^{\text{f}}({\text{γ}}_{i,r}\;+\;{\text{γ}}_{r}^{g})u_{\text{i}}\left(\text{t}-\text{r}\right)\;\\ \text{ + }\sum_{\text{q=1}}^{g}\sum_{r\text{=1}}^{h}\left(\tau_{i,q,r}+\tau_{q,r}^{g}){\text{η}}_{i}\left(t-\text{q}\right){\text{u}}_{i}\left(t-\text{r}\right)\text{​}+\text{​}{\text{ζ}}_{i}(\text{t}\right)\; \end{array}$$

where η_*i*_(t), A, Φ, q, a, u, γ, r, f, τ, g, h, and ζ_*i*_ are defined as in Equation (5), g represents (p,p)-dimensional matrices of regression coefficients for group-level connections, and i represents (p,p)-dimensional matrices of regression coefficients for individual-level connections. GIMME is fit in a data-driven manner. The structures of the group A, Φ, γ, and τ matrices are determined by iteratively freeing for all individuals the significant connection that modification indices show will most improve model fit for a specified criterion of the sample (usually 75%), and then structures of the individual A, Φ, γ, and τ matrices are determined by iteratively freeing connections that modification indices show will most improve model fit for an individual. GIMME is a state-of-the-art approach for mapping neural connectivity. In large scale simulations, it outperformed other undirected functional, directed functional, and effective connectivity mapping techniques, including dynamic causal modeling (Smith et al., 2011; Gates and Molenaar, 2012).

Thus, uSEM, euSEM, and GIMME constitute the uSEM family of approaches because are all structural VARs, which map contemporaneous and lagged connections, fit directly to the data. uSEMs map individual-level connections among ROIs, euSEMs consider how task conditions affect individual-level (linear and bilinear) connections among ROIs, and GIMME implements uSEMs or euSEMs at the group-level allowing individual-level heterogeneity. uSEM, euSEM, and GIMME are not simply forms of SEMs, which only map contemporaneous connections, or standard VARs, which only map lagged connections.

Despite its accuracy, the uSEM family of approaches has only been evaluated in simulations and applied to fMRI data sets using first order lags (Gates et al., 2010, 2011; Hillary et al., 2011; Gates and Molenaar, 2012; Beltz et al., 2013; Hillary et al., 2014; Karunanayaka et al., 2014; Nichols et al., 2014; Grant et al., 2015; Yang et al., 2015). This may have been appropriate for applications concerning task effects (Hillary et al., 2011, 2014; Grant et al., 2015; Yang et al., 2015). Tasks temporally direct neural processes (e.g., by increasing difficulty with repeated assessments or by deactivating the default mode; Poldrack, 2000; Raichle et al., 2001), emphasizing contemporaneous connections among ROIs, especially for fast event-related designs in which multiple stimuli are presented within a single HRF iteration or functional volume. Moreover, some applications utilized functional data with 3s TRs now considered to be long (Beltz et al., 2013). Long TRs provide measurements containing weaker auto- and cross-correlations than short TRs, due in part to the nature of the HRF (Arbabshirani et al., 2014). Therefore, it unclear whether directed functional connectivity maps generated using first order models from the uSEM family of approaches sufficiently capture all sequential dependencies in fMRI data, particularly in resting state data collected at currently-used lags (e.g., 2s TRs).

### Current study

The aim of the current study was to present and apply an a posteriori model validation procedure for the temporal order of directed functional connectivity maps estimated with the uSEM family of approaches (i.e., uSEM, euSEM, and GIMME). The procedure included conducting established white noise tests of the maps' one-step-ahead prediction errors (Box and Jenkins, 1970) then updating the maps when tests indicated that the model order was insufficient for capturing all sequential dependencies in the data. We applied the validation procedure to several different blood oxygen level-dependent (BOLD) fMRI data sets to demonstrate its utility across scenarios: A single-subject simulated data set with second order lags but without external input (analyzed with uSEM), a single-subject empirical data set with external input (analyzed with euSEM), and a 32-subject empirical resting state data set without external input (analyzed with GIMME). The first two data sets were generated or selected to showcase the a posteriori model validation procedure in the context of directed functional connectivity mapping, a context in which it has not been previously applied. The final, 32-subject empirical data set was not pre-selected, and thus, provided a test of the validation procedure as well as a realistic demonstration of the prevalence of higher order lags in fMRI resting state data. Due to the characteristics of the data set (i.e., resting state fMRI data collected at a 2s TR), we expected that the directed functional connectivity maps for some subjects would require lags greater one.

## Materials and methods

One simulated data set was generated and two empirical data sets were obtained, preprocessed, and had ROIs extracted prior to being analyzed for directed functional connectivity of the first order using an approach within the uSEM family (i.e., uSEM, euSEM, or GIMME). Directed functional connectivity maps were then submitted to a posteriori model validation using white noise tests to determine whether higher order lags were required. If so, then maps were altered according to the results of the white noise tests, and the process iterated (i.e., model fitting, white noise testing, etc., was repeated) until the maximum modeled order was deemed sufficient for capturing all sequential dependencies in the data.

### Simulated data

**Figure 1:**
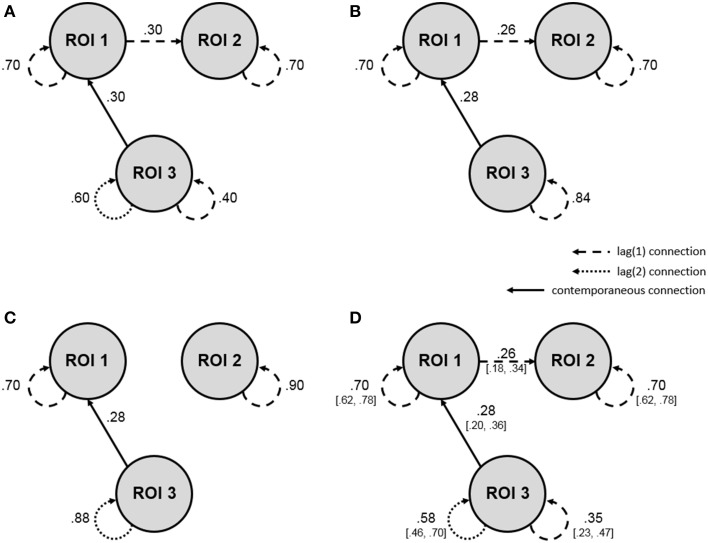
**Single-subject simulation showing how the a posteriori model validation procedure can be applied to fMRI data analyzed for directed functional connectivity using the uSEM family of approaches**. Dashed lines reflect first order connections, dotted lines reflect second order connections, solid lines reflect contemporaneous connections, and values show connection strengths (i.e., beta-weights; all are significant at *p* = 0.05). **(A)** Simulation for a single subject according to a second order uSEM with 3 ROIs and 200 time points, with beta-weights showing true parameter values. **(B)** Map resulting from a first order data-driven uSEM that did not contain white noise residuals. **(C)** Map resulting from a second order data-driven uSEM that did not contain white noise residuals. **(D)** Final map with white noise residuals resulting from the freeing of two additional confirmatory parameters, as indicated by modification indices from the second order data-driven uSEM white noise test. The final map precisely recovered the simulated map, with true parameter values falling within the bracketed 95% confidence intervals of the estimated beta-weights. See **Table 1** for model fit and white noise test results.

A second order single-subject BOLD fMRI data set without external input was simulated according to a uSEM, with the zero mean process innovation having a covariance matrix equal to the identity matrix. It contained 3 ROIs and 200 measurements, with a pattern of connections specified as in Figure 1A. Each ROI had a first order autoregressive component, and the third ROI had an additional second order autoregressive component. Activity in the first ROI was contemporaneously predicted by activity in the third ROI and predicted second ROI activity at a lag of one.

### Empirical data

Two BOLD fMRI data sets were utilized. Different preprocessing and ROI definition procedures were used due to differences between the data sets (e.g., task procedures), to maintain consistency with past work (e.g., Wilson et al., 2012; Nichols et al., 2014), and to showcase the widespread applicability of the validation procedure.

#### Single-subject with external input

The first data set was from a single subject completing a verbal working memory task. Data from this subject were included in prior reports on the effects of smoking on reward circuitry, according to standard GLM (Wilson et al., 2012) and directed functional connectivity analyses (Nichols et al., 2014). The data were selected for presentation here because they showcase how the validation procedure can be applied to task-related data.

##### Participant

Study inclusion criteria specified that this male subject be a right-handed, regular cigarette user between 18 and 45 years of age who smoked between 15 and 40 cigarettes a day for the past 2 years (Wilson et al., 2012).

##### Procedure

Detailed testing procedures are described elsewhere (Wilson et al., 2012; Nichols et al., 2014). Briefly, the subject refrained from smoking for the 12 h preceding the neuroimaging session, verified with expired air carbon monoxide levels. During the session, the subject provided structural and functional MRI data via a 3-Tesla head-only Siemens Allegra magnet with a standard head coil. Structural MRI data included a 40 slice oblique-axial anatomical series (3.125 × 3.125 × 3.0 mm voxels) acquired parallel to the anterior commissure-posterior commissure plane using a standard T2-weighted pulse sequence and a high-resolution (1 mm^3^ voxels) three-dimensional structural volume using a magnetization-prepared rapid gradient-echo (MPRAGE) sequence. Functional images (*N* = 142) included a one-shot echo-planar imaging (EPI) sequence (*TR* = 2000 ms, *TE* = 25 ms, FOV = 200 mm, flip angle = 79°) acquired in the same plane as the 40-slice anatomical series with coverage limited to the 38 center slices during a verbal working memory task. Specifically, the event-related *n*-back task consisted of six 36-s task blocks during which 12 (of 18) randomly-selected English letters individually appeared (each 500 ms) between fixation crosses (each 2500 ms) and two 36-s rest blocks. In each of the three control (0-back) blocks, the subject pressed a button each time he saw the letter X (i.e., target). In each of the three experimental (3-back) blocks, the subject pressed a button each time the target letter matched a letter he saw exactly three items ago. The target letter required a button press 33% of the time.

##### Preprocessing

Standard preprocessing was conducted, including motion correction, slice-timing correction, drift adjustment, and alignment to Montreal Neurologic Institute (MNI) space using the subject's structural images and a six-parameter rigid-body automated registration algorithm. The data were globally mean-normalized and smoothed using a three-dimensional Gaussian filter (8-mm full width at half maximum).

##### ROI definition

Task-related ROIs were identified using a standard two-level random-effects GLM implemented with AFNI (Cox, 1996). Individual-level parameter estimates for each version of the n-back task were obtained from a GLM, and then a group-level paired *t*-test was used to determine which brain regions displayed an effect of memory load (i.e., 3-back > 0-back) using a voxel-wise significance threshold of *p* < 1 × 10-18 and a spatial extent threshold of 10 contiguous voxels. BOLD time series were extracted from each of the identified ROIs for further analyses: dorsal anterior cingulate cortex (ACC), right and left dorsolateral prefrontal cortex (R/L DLPFC), right and left lateral premotor cortex (R/L LPM), and right and left inferior parietal lobule (R/L IPL). The time series had a length of 142.

#### Multi-subject resting state

The second empirical data set was from a 32-subject sample during resting state. Data were collected as part of a larger neuroimaging study on the effects of alcohol use on inhibitory and reward processing in university students. This data set was not previously analyzed and came from a sample (i.e., college students) commonly used in fMRI research; thus, it not only provides a demonstration of how a posteriori model validation for the temporal order of directed functional connectivity maps can be implemented at the group-level, but it also serves as an a priori test of the prevalence of higher order lags in typical resting state fMRI data.

##### Participants

Subjects were 32 second or third year university students (18 female), aged 20–21 years. They were recruited from an intervention study on the effects of parental communication on alcohol use behaviors in college (Varvil-Weld et al., 2012, 2014; Turrisi et al., 2013), with most subjects (69%) receiving the intervention after neuroimaging.

##### Procedure

Subjects participated in a 2-h neuroimaging and behavioral data collection session. During neuroimaging, subjects provided structural and functional MRI data via a 3-Tesla Siemens Trio magnet with a standard head coil. Structural MRI data included a high-resolution (1 mm^3^ voxels) three-dimensional volume acquired using an MPRAGE sequence. Functional images included an EPI sequence (*TR* = 2000 ms, *TE* = 25 ms, FOV = 240 mm, flip angle = 80°) obtained with an interleaved acquisition of 39 slices with 3 mm^3^ voxels during resting state. Subjects were instructed to relax with their eyes closed, trying not to think of anything in particular or fall asleep.

##### Preprocessing

Standard preprocessing was conducted in FSL (Jenkinson et al., 2012), including removal of the first four volumes, motion correction via removal of six movement vectors from the data (X, Y, Z, pitch, yaw, roll), slice-timing correction, non-brain removal, spatial smoothing using a Gaussian kernel (6-mm full width at half maximum), grand-mean intensity normalization by a single multiplicative factor, highpass temporal filtering (sigma = 100.0 s), and registration to standard MNI space. Additional physiological noise (e.g., cardiac and respiratory signal) was removed from the functional data by covarying BOLD signal from a white matter (MNI central coordinate: *x* = 26, *y* = −12, *z* = 35) and a cerebral spinal fluid (MNI central coordinate: *x* = 19, *y* = −33, *z* = 18) ROI (Chang and Glover, 2009; Murphy et al., 2013).

##### ROI definition

Four ROIs reflecting the default mode network (DMN) were selected a priori using central MNI coordinates from past resting state research (Biswal et al., 2010; Van Dijk et al., 2010): posterior cingulate cortex (PCC; *x* = −5, *y* = −49, *z* = 40), medial prefrontal cortex (MPFC; *x* = −1, *y* = 47, *z* = −4), right lateral parietal lobule (R LP; *x* = 46, *y* = −62, *z* = 32), and left lateral parietal lobule (L LP; *x* = −45, *y* = −67, *z* = 36). Size of DMN (and white matter and cerebral spinal fluid) ROIs depended upon brain volume, with the sample median brain volume of 1297 cm^3^ having ROIs with a 6.5 mm radius and ROIs for all other brain volumes being a linear proportion of this. BOLD time series were extracted from ROIs for further analyses. Final time series had a length of 160.

### Data analysis plan

Data were analyzed in two steps. First, each data set was submitted to directed functional connectivity analyses using the appropriate technique from the uSEM family (i.e., uSEM, euSEM, or GIMME). Second, results from the connectivity analyses were submitted to the a posteriori model validation procedure, consisting of a potentially iterative process of white noise testing and estimating higher order directed functional connectivity maps based on results from the white noise tests.

#### Directed functional connectivity analyses

The three data sets were submitted to directed functional connectivity analyses using a first order uSEM, euSEM, or GIMME model. Analyses were conducted in LISREL (Jöreskog and Sörbom, 1992) and Matlab® (Mathworks, 2013) and GIMME is publicly available at http://www.nitrc.org/projects/gimme. (Note that analyses can be conducted in any structural equation modeling software that conducts Lagrange Multiplier testing, such as lavaan or OpenMx, (Boker et al., 2011; Rosseel, 2012); a version of GIMME that runs in R using lavaan is available at https://cran.r-project.org/web/packages/gimme). The simulated data set was analyzed with uSEM (Gates et al., 2010) because it contained data from a single subject and no external input. The single-subject empirical data set was analyzed with euSEM (Gates et al., 2011) because it contained external input. A single vector specifying the presence or absence of the experimental (i.e., 3-back) task condition convolved with a double gamma function to approximate the HRF was used as input. The multi-subject empirical data set was analyzed with uSEM implemented via GIMME (Gates and Molenaar, 2012) in order to determine the group- (and individual-) level structure of the data. The grouping criterion was set to 75%; thus, a connection between two DMN ROIs was required to be significant for at least 24 (of 32) subjects in order to be estimated for everyone in the sample. All models were evaluated with alternative fit criteria at the individual-level, and models were accepted if two of four alternative indices indicated excellent fit (Brown, 2006): RMSEA ≤ 0.05, SRMR ≤ 0.05, CFI ≥ 0.95, NNFI ≥ 0.95. This assessment of model fit has yielded excellent results in simulation studies (Gates et al., 2010, 2011; Gates and Molenaar, 2012).

#### A posteriori model validation tests

**Figure 2:**
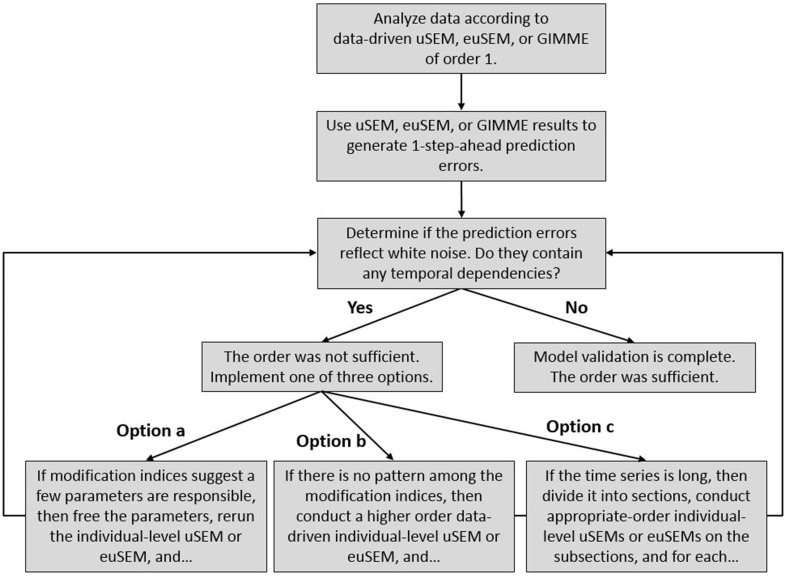
**A posteriori model validation procedure for the temporal order of directed functional connectivity maps generated by the uSEM family of approaches (i.e., uSEM, euSEM, GIMME), consisting of white noise testing of prediction errors and the application of decision criteria for generating higher order individual-level maps, when necessary**.

The validation procedure is summarized in Figure 2. Even though white noise testing of one-step-ahead prediction errors is well-established (Box and Jenkins, 1970), it has not been applied to the analysis of neuroimaging data, particularly directed functional connectivity analyses. Nor has it been combined with decision criteria (options a through c in Figure 2) for amending model results based upon modification indices.

To conduct a posteriori model validation, directed functional connectivity was determined via a first order analysis from the uSEM family of approaches, and then the resulting individual-level models were used to generate one-step-ahead prediction errors (Box and Jenkins, 1970). For example, prediction errors for a uSEM(1) are estimated as:

(7) $$\begin{array}{l} {\hat{\text{ζ}}}_{i}(\text{t})={\text{ η}}_{i}(\text{t})-\hat{\text{A}}{\text{η}}_{i}(\text{t})-{\hat{\Phi }}_{1}{\text{η}}_{i}(\text{t}-1), \end{array}$$

where ${\hat{\zeta }}_{i}$ is the estimated p-variate process innovations, η_*i*_(t) is defined as in Equation (4); $\hat{\text{A}}$ is the estimated (p,p)-dimensional matrix of contemporaneous regression coefficients, and ${\hat{\Phi }}_{1}$ is the estimated (p,p)-dimensional matrix of regression coefficients at lag 1.

White noise tests were then carried out in LISREL (Jöreskog and Sörbom, 1992) by fitting a high order (3) VAR to ${\hat{\zeta }}_{i}$ by means of the block Toeplitz method (Molenaar, 1985) and testing whether a model specifying that all lagged regression coefficients in the VAR(3) not significantly differ from zero fit the estimated errors, according to at least two of four alternative fit indices (Brown, 2006): RMSEA ≤ 0.05, SRMR ≤ 0.05, CFI ≥ 0.95, NNFI ≥ 0.95. Annotated LISREL syntax for the white noise test is presented in the Appendix. If the model fit well, then a posteriori model validation was complete, and the first order model was selected. If the model did not fit well, then one of the three options noted in Figure 2 could have been implemented: (option a) when modification indices from the white noise test indicated a few, (often) second order parameters would improve model fit, those parameters could be freed in a confirmatory uSEM or euSEM; (option b) when there was no clear pattern among the modification indices, an exploratory second order individual-level uSEM or euSEM could be fit to the data; (option c) when the time series was long and there was reason to think it might reflect substantively different neural processes (e.g., task conditions), the data could be subdivided and second order uSEMs or euSEMs fit to some of the subsections. After implementing one of the options, the validation procedure was repeated using the one-step-ahead prediction errors from the new model. These procedures could be repeated for orders higher than two.

It is important to highlight that the validation procedure was conducted on individual-level models; this is obvious for the single-subject simulated and empirical data sets, but perhaps not for the multi-subject data set analyzed with GIMME. As stated above, GIMME implements uSEMs (or euSEMs) of the first order, and each subject's GIMME results include group-level connections (i.e., a set of ROI relations freed in all subjects' maps) and individual-level connections (i.e., a set of ROI relations freed only for a given subject). In the current multi-subject analyses, when the a posteriori model validation procedure indicated that a first order model was not sufficient for capturing all sequential dependencies in a subject's data, confirmatory (i.e., option a) or exploratory (i.e., option b) second order uSEMs were conducted on the individual-level data after estimating the GIMME group-level model. In other words, the null model used in second order individual-level uSEM analyses was the group-level model. This maintained the group-level structure of the data, while allowing for individual-level connections to capture higher order temporal processes.

## Results

Results presented below confirmed expectations. After fitting directed functional connectivity maps and applying the a posteriori model validation procedure described in Figure 2, we determined that second order lags were required in the uSEM map of single-subject simulated data and in 14 out of 32 individual-level GIMME maps of empirical resting state data, but not in the euSEM map of single-subject empirical data containing external input.

### Simulated data analyzed with uSEM

A first order data-driven uSEM was first fit to the simulated data. The resulting map and fit indices are shown in Figure 1B and the top of the second column of Table 1, respectively. The model fit the data well, but did not have white noise residuals, according to fit indices shown in the bottom of the second column of Table 1. Modification indices from the white noise test did not display a clear pattern, so a second order data-driven uSEM was next fit to the data, that is, option b from Figure 2 was applied. The resulting map is shown in Figure 1C; it fit the data well, but did not have white noise residuals (see the third column of Table 1). Modification indices from the white noise test indicated that two first order parameters (i.e., an autoregressive connection for ROI 3 with MI = 31.29 and a connection from ROI 1 to ROI 2 with MI = 31.52) were responsible for the misfit, so a second order confirmatory uSEM, freeing these two additional parameters was fit, that is, option a from Figure 2 was applied. The confirmatory map is shown in Figure 1D; it fit the data well and had white noise residuals (see the final column of Table 1). Moreover, the model recovered all and only the true parameters from the simulation, with the true connection values falling within the bracketed 95% confidence intervals shown in Figure 1D.

**Table 1:** **Model fit indices and white noise test results for the series of uSEM models fit to the single-subject simulated data set**.

	**Data-driven uSEM(1)**	**Data-driven uSEM(2)**	**Confirmatory uSEM(2)**
**MODEL FIT**			
*χ^2^* (*p*)	18.91(p < 0.01)	99.26(p < 0.001)	30.89(p < 0.01)
*df*	7	17	15
RMSEA	0.09	0.16	0.07
SRMR	0.03	0.04	0.01
CFI	0.99	0.97	0.99
NNFI	0.98	0.93	0.98
**WHITE NOISE TEST**			
*χ^2^* (*p*)	269.98(p < 0.001)	192.00(p < 0.001)	64.07(p > 0.05)
*df*	72	72	72
RMSEA	0.12	0.09	0.00
SRMR	0.16	0.11	0.06
CFI	0.38	0.38	1.00
NNFI	0.43	0.43	1.00

*uSEM, unified structural equation model; df, degrees of freedom; RMSEA, root mean squared error of approximation; SRMR, standardized root mean residual; CFI, confirmatory fit index; NNFI, non-normed fit index. Model fit and white noise test accepted if two of four indices indicate excellent fit: RMSEA ≤ 0.05, SRMR ≤ 0.05, CFI ≥ 0.95, NNFI ≥ 0.95*.

### Single-subject empirical data analyzed with euSEM

A first order data-driven euSEM was fit to the single-subject empirical data containing external input. The map fit the data well, with all four alternative indices indicating excellent fit: χ^2^_(106)_ = 136.15, *p* < 0.05, RMSEA = 0.05, SRMR = 0.03, CFI = 0.99, NNFI = 0.99. The map also contained white noise residuals, as a model specifying that all lagged regression coefficients in a VAR(3) of the one-step-ahead prediction errors not significantly differ from zero fit well: χ^2^_(378)_ = 480.96, *p* < 0.001, RMSEA = 0.03, SRMR = 0.12, CFI = 0.99, NNFI = 0.99. Thus, an euSEM of the first order was sufficient for the data.

**Figure 3:**
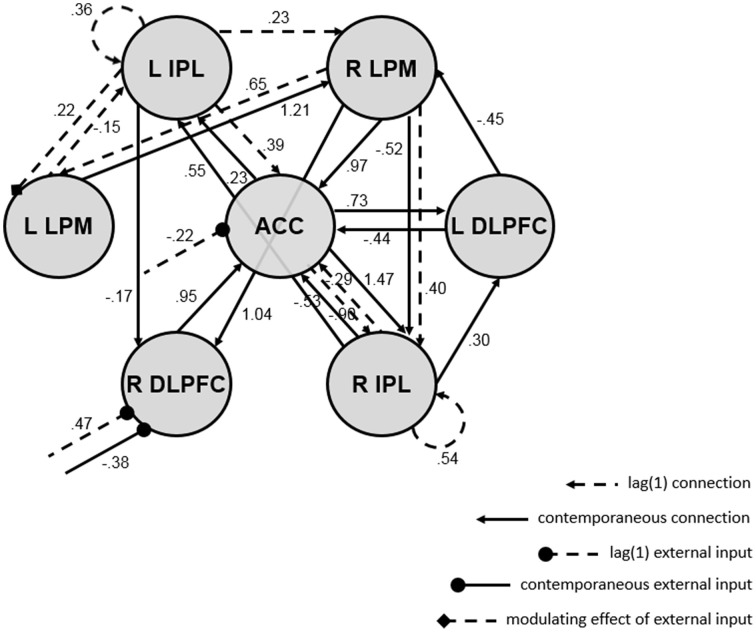
**Directed functional connectivity map from euSEM analysis of single-subject empirical data with external input (i.e., a vector indicating the experimental condition of a verbal working memory task)**. Dashed lines reflect first order connections, solid lines reflect contemporaneous connections, arrows reflect ROI connections, circular endpoints reflect direct effects of the task, diamond endpoints reflect modulating effects of the task, and values show connection strengths (i.e., beta-weights; all are significant at *p* = 0.05). The map fit the data well and had white noise residuals; see fit statistics in the text. ACC, anterior cingulate cortex; R/L DLPFC, right/left dorsolateral prefrontal cortex; R/L LPM, right/left lateral premotor cortex; R/L IPL, right/left inferior parietal lobule.

The resulting map is shown in Figure 3, and it depicts several interesting results. First, there were more contemporaneous than lagged connections in the network, and only two autoregressive components (for the right and left IPL). Second, the 3-back task impacted network connectivity. The presence (vs. absence) of the task was associated with the presence of a lagged connection from the left IPL to the left LPM, lagged decreases in ACC activity, and lagged increases but contemporaneous decreases in right DLPFC activity, perhaps reflecting a feedback mechanism. Third, the ACC and right IPL appear to be hubs of the network, as they had the highest degree of all ROIs (i.e., the most incoming and outgoing connections with other ROIs).

### Multi-subject empirical data analyzed with GIMME

**Figure 4:**
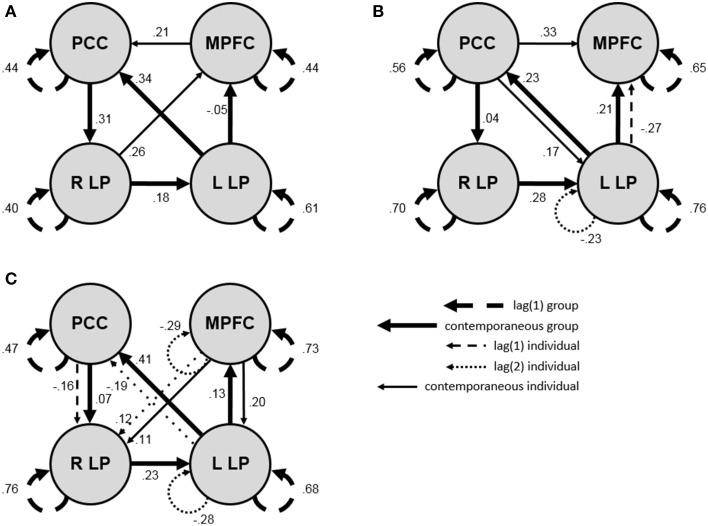
**Exemplar final directed functional connectivity maps from GIMME analysis and a posteriori model validation of a 32-subject resting state data set**. Thick lines reflect group-level connections, thin lines reflect individual-level connections, dashed lines reflect first order connections, dotted lines reflect second order connections, solid lines reflect contemporaneous connections, and values show connection strengths (i.e., beta-weights; all are significant at *p* = 0.05 except for group paths ≤ |0.13|). **(A)** Final map for a subject requiring only the lag one and contemporaneous connections estimated in GIMME; the map fit the data well [χ^2^_(12)_ = 17.40, *p* > 0.05, RMSEA = 0.05, SRMR = 0.04, CFI = 0.99, NNFI = 0.98] and contained white noise residuals [χ^2^_(126)_ = 136.94, *p* > 0.05, RMSEA = 0.00, SRMR = 0.10, CFI = 0.98, NNFI = 0.98]. **(B)** Final map for a subject requiring second order connections, determined via a confirmatory second order uSEM, which fit the data well [χ^2^_(26)_ = 54.54, *p* < 0.001, RMSEA = 0.08, SRMR = 0.04, CFI = 0.98, NNFI = 0.96] and contained white noise residuals [χ^2^_(126)_ = 135.08, *p* > 0.05, RMSEA = 0.00, SRMR = 0.07, CFI = 0.98, NNFI = 0.98]. **(C)** Final map for a subject requiring second order connections, determined via a data-driven second order uSEM, which fit the data well [χ^2^_(23)_ = 17.35, *p* > 0.05, RMSEA = 0.00, SRMR = 0.02, CFI = 1.00, NNFI = 1.00] and contained white noise residuals [χ^2^_(126)_ = 72.64, *p* > 0.05, RMSEA = 0.00, SRMR = 0.06, CFI = 1.00, NNFI = 1.00]. PCC, posterior cingulate cortex; MPFC, medial prefrontal cortex; R/L LP, right/left lateral parietal lobule.

GIMME was fit to the multi-subject empirical data set, implementing uSEMs of the first order to generate a final map for each subject that contained group- and individual-level connections. The structure of the group-level map (with connections that were fit for all subjects, allowing for variations in the magnitude and direction of the connections across subjects) is shown by the thick lines in the exemplar maps in Figure 4; notice that these group-level connections are present in each of the maps. They indicate that there was a prominent group-level DMN, with eight connections among the four ROIs (i.e., a first order autoregressive connection for each ROI and four contemporaneous connections between ROIs). After adding individual-level lag one and contemporaneous connections to the group-level map, all subjects had final maps that fit their data well, with mean fit indices across subjects of χ^2^_(11)_ = 14.75, *p* > 0.05, RMSEA = 0.03, SRMR = 0.03, CFI = 1.00, and NNFI = 0.99.

The final map for each subject was then submitted to a white noise test to determine whether a lag of one was sufficient for capturing all sequential dependencies the data. For 18 of the 32 (56%) subjects, a first order uSEM was sufficient; thus, the final maps for these subjects were directly output by GIMME. For the remaining 14 subjects, a second order uSEM was required. This was determined by generating confirmatory second order uSEMs based on the modification indices from the first order white noise tests for seven subjects (i.e., option a in Figure 2), and by conducting a second order data-driven search for individual-level connections (including the estimation of all group-level connections) for seven subjects (i.e., option b in Figure 2). Figure 4 shows final exemplar directed functional connectivity maps for a subject in each of the three scenarios (i.e., first order map, second order confirmatory map, second order data-driven map). Figure 4A shows the map for a subject who required only first order and contemporaneous connections; the group-level map and two additional individual-level contemporaneous connections were freed in GIMME. Figure 4B shows a map for a subject who required a confirmatory second order connection; the group-level map and three individual-level lag one and contemporaneous connections were freed in GIMME, and white noise tests indicated that a second order autoregressive connection for the left LP with MI = 6.16 was necessary to account for all sequential dependencies in the data. Figure 4C shows a map for a subject who required a second order data-driven search for individual-level connections because the freeing of confirmatory parameters indicated by modification indices from the white noise test failed to yield acceptable results; in the data-driven search, the group-level map and seven individual-level connections (four second order, one first order, and two contemporaneous) were freed. Final maps for all subjects after GIMME analysis and temporal validation fit the data well, with mean fit indices across subjects of χ^2^_(17)_ = 24.75, *p* > 0.05, RMSEA = 0.04, SRMR = 0.03, CFI = 0.99, and NNFI = 0.98. The final maps also generated white noise residuals, with mean fit indices across subjects for models specifying that all lagged regression coefficients in a VAR(3) of the one-step-ahead prediction errors not significantly differ from zero of χ^2^_(126)_ = 126.76, *p* > 0.05, RMSEA = 0.01, SRMR = 0.08, CFI = 0.98, and NNFI = 0.99.

## Discussion

Seldom is a posteriori model validation for the temporal order of directed functional connectivity maps conducted in the analysis of neuroimaging data. Despite concerns about autoregressive processes confounding GLM results (Friston et al., 2000; Woolrich et al., 2001; Penny et al., 2003) and recent interest in the effect of auto- and cross-correlations on undirected functional connectivity map parameters (Christova et al., 2011; Kaneoke et al., 2012; Arbabshirani et al., 2014), little attention has been paid to the oft violated assumption of independence among residuals in directed functional connectivity maps. The goal of this paper was to fill that knowledge gap, calling attention to the issue and addressing it via white noise testing of one-step-ahead prediction errors (Box and Jenkins, 1970) generated from uSEM family connectivity maps (Gates et al., 2010, 2011; Gates and Molenaar, 2012), and map revision informed by newly-developed decision criteria based upon modification indices from the white noise test results (Sörbom, 1989; Lütkepohl, 2005). Model fitting, white noise testing, and map revision (when necessary) was applied to single-subject simulated, single-subject task-related, and multi-subject resting state fMRI data.

Findings revealed that the a posteriori model validation procedure (i.e., white noise testing combined with decision criteria) is accurate and can successfully be applied to simulated and empirical neuroimaging data. A single-subject simulation was conducted to showcase how the procedure applies to fMRI data. The data set contained contemporaneous, first order, and second order connections among three ROIs. White noise tests indicated that a first order model was insufficient, as sequential dependencies were present among the residuals, but that a second order model was sufficient. This second order model recovered all and only the true parameters. Additional simulations were deemed unnecessary because the validation procedure has been substantiated and widely-used in other contexts, such as econometrics (Box and Jenkins, 1970; Akaike, 1974; Lütkepohl, 2005).

Single-subject empirical data containing a vector denoting the experimental condition of a verbal working memory task were analyzed to demonstrate how the validation procedure applies to data with external input. White noise tests indicated that a first order model was sufficient; contemporaneous and lag one connections accounted for all sequential dependencies among the seven ROIs and reflected direct and modulating effects of the task on the network. Moreover, there were fewer lagged than contemporaneous connections in the network, including only two of seven possible first order autoregressive components, a noteworthy finding because more lagged than contemporaneous connections are possible in euSEM maps (cf. Gates et al., 2011). The sufficiency of the first order model and the prominence of contemporaneous connections was expected: The data were selected to highlight how tasks can direct neural processes (Poldrack, 2000; Raichle et al., 2001), and thus, how task-related data may be less likely than resting state data to contain higher order lags, as neural processes occur proximate to external stimuli, especially in fast event-related designs. Along with other data showing that instantaneous connections are more closely linked to neural structural connectivity than are lagged connections (Alonso-Montes et al., 2015), these results suggest that contemporaneous connections may be of greatest substantive interest to neuroscientists. Yet, proper modeling of the temporal order of neuroimaging data with lagged connections is required for contemporaneous connections—and the inferences drawn from them—to be accurate. It is paramount to further explore these notions in future work.

Multi-subject resting state data were analyzed to exhibit how the validation procedure applies at the group-level. These results were of primary interest because they served to make both a methodological point about the value of white noise testing and the decision criteria, and to make a substantive point about the prevalence of higher order lags in the widely-studied DMN. White noise tests of final individual-level maps, which contained group- and individual-level contemporaneous and first order connections among four ROIs, revealed white noise residuals for 56% of the subjects; thus, first order maps were insufficient for 44% of the subjects. For the latter, second order maps were obtained via modification indices: Confirmatory second order individual-level connections were added or all individual-level connections were re-estimated in a data-driven procedure, allowing lag two connections to be freed. These results highlight the prevalence of higher order lags in resting state fMRI data, and suggest that connectivity mapping techniques that do not account for contemporaneous, lag one, and lag two connections among ROIs may be inaccurate. Although the optimal preprocessing pipeline for resting state data is controversial (see e.g., Fox et al., 2009; Murphy et al., 2009), it seems unlikely that the procedures employed here systematically influenced the results. Preprocessing procedures were moderate (e.g., inclusion of six motion and two physiological noise—but no global signal—regressors), so as not to remove too little or too much variance in the BOLD data. Findings also varied at the individual-level; there would be relative uniformity across subjects if preprocessing was responsible for the results.

Findings are somewhat contrary to a recent report on the impact of autocorrelation in undirected functional connectivity mapping in which it was argued that corrections for autocorrelations do not affect results of hypothesis tests (e.g., the significance of connections) for resting state data (Arbabshirani et al., 2014). It is unlikely that procedural differences between the recent report and the current study (e.g., undirected vs. directed connectivity analyses, estimation of full models vs. sparse models, consideration of autocorrelation vs. auto- and cross-regression, respectively) fully account for the opposing inferences. The simulation presented here shows that hypothesis tests can be affected by misspecification of the temporal order used in connectivity analyses: The true second order autoregressive component for the third ROI was absent when a lag one model was implemented, resulting in the artificial inflation of the first order autoregressive parameter for this ROI. Also, the multi-subject application presented here shows that second order connections must be modeled in order to obtain white noise residuals for nearly half of subjects' resting state data. Inferences about group-level DMN connectivity would have been faulty had they been made from lag one maps instead of lag two maps for these subjects, as the structure (i.e., presence and absence) of connections—not just the magnitude of the connections—differed between lags.

All directed functional connectivity analyses presented here were estimated using the uSEM family of approaches. The single-subject simulated data were analyzed with uSEM (Gates et al., 2010), the single-subject empirical data with external input were analyzed with euSEM (Gates et al., 2011), and the multi-subject resting state data were analyzed with GIMME (Gates and Molenaar, 2012). The uSEM family was used because the constituent models accurately estimate both contemporaneous and lagged connections, can accommodate task-related input, provide group- and individual-level maps, and have been shown to outperform competing models in large scale simulations (Smith et al., 2011; Gates and Molenaar, 2012). Nonetheless, results are expected to generalize to other directed functional connectivity analysis approaches (e.g., SEM and VAR) and even to undirected functional connectivity analysis approaches (e.g., seed-based Pearson correlations and independent components analysis). In fact, it is possible, even likely, that implementing the a posteriori model validation procedure on connectivity maps obtained with suboptimal analysis approaches will lead researchers to more optimal analysis approaches. For example, if residuals from a directed functional connectivity map obtained via SEM are not white noise, then lagged connections must be added to the model; a logical way to accomplish this would be through the use of a structural VAR, such as uSEM.

All data presented here were obtained from BOLD fMRI, as fMRI is currently the most widely-used neuroimaging tool for the study of human neural connectivity (Smith, 2012), but the connectivity analyses and a posteriori validation procedures are expected to generalize to data obtained from other neuroimaging methods, potentially with varying patterns of results. For example, it is highly unlikely that first order directed functional connectivity maps would be sufficient for electroencephalogram (EEG) data because they have much higher temporal resolution than do fMRI data; the short measurement intervals lead to high and higher-order sequential dependencies. Thus, EEG map residuals would be expected to reflect white noise only after lags of an order greater than two (i.e., the maximum required for the fMRI data presented here) were incorporated into uSEM, euSEM, and GIMME models.

There are alternatives to some procedures implemented in the current work, and they should be considered when interpreting findings and designing future studies. First, the HRF could be incorporated into the analyses in ways other than those employed here. For task-related data, we convolved the vectorized task condition with the double gamma function, but other HRF approximations could be used. The BOLD data (in both task-related and resting state data) could also be deconvolved allowing for different HRF parameters in different brain regions and subjects (e.g., Handwerker et al., 2004; Wu et al., 2013). The implications of different HRF implementations for directed functional connectivity results, particularly those generated from the uSEM family of approaches with their lagged effects and group- and individual-level maps, is an important area for future work.

Second, the multi-subject data set was analyzed with GIMME, which implements uSEMs (or euSEMs) of the first order; consequently, all identified group-level connections were contemporaneous or lag one, and all higher order (in this case, lag two) temporal dependencies were accounted for by individual-level connections. Although GIMME could be modified to identify group-level connections at lags greater than one, there was no evidence that this was necessary for the current data set: Lag two connections were not present for the sample criterion (i.e., the 75% of subjects required to have a significant connection in order to free the connection at the group-level). There is certainly an opportunity for future work in this area, though, as it is possible to imagine data sets for which higher order group-level connections may be needed (e.g., resting state fMRI data collected at 1s TRs or EEG data).

Third, temporal lags were validated a posteriori. This is consistent with traditional white noise testing (Box and Jenkins, 1970), and with hallmark work on the selection of VAR model orders based on Akaike Information Criteria generated after model fitting (Akaike, 1974). It also facilitates the use of modification indices for map refinement because the indices are generated after model fitting (Lütkepohl, 2005), and the estimation of a group-level map in GIMME because the data-driven search is based on identical parameter spaces (i.e., starting with null models) for all subjects (Gates and Molenaar, 2012). Nonetheless, it is possible to gather information about model order a priori using the autocorrelation function, for example. Future work on the practicality and effectiveness of such an approach may be beneficial.

Fourth, decision criteria implemented here for the refinement of insufficiently ordered directed functional connectivity maps were based on modification indices (i.e., Lagrange Multiplier tests that can be obtained from SEM software packages, such as LISREL, lavaan, or OpenMx; Sörbom, 1989; Jöreskog and Sörbom, 1992; Boker et al., 2011; Rosseel, 2012). Alternatives, such as dividing the BOLD time series into subsections for independent analysis (i.e., Figure 2, option c), were not necessary. This was likely a byproduct of the data sets (e.g., they had relatively short empirical time series) and computational power of the modification indices. There are circumstances, however, for which time series division may be highly applicable, particularly for data sets with external input. Examples include data sets with task-related non-stationarity and data sets from task designs with long blocks.

In conclusion, directed functional connectivity mapping requires a posteriori model validation of the temporal order. Even when maps provide a good fit to neuroimaging data according to usual goodness-of-fit criteria, the residuals may still contain sequential dependencies, violating model assumptions. Validation can be accomplished via white noise testing of prediction errors combined with decision criteria for refining the maps based on modification indices from the tests. Application of the validation procedure to fMRI data demonstrated that it leads to accurate results in simulated data and that it can be applied to empirical task-related data. Most importantly, it showcased that higher order (i.e., greater than one) lags must be considered when analyzing resting state data, as maps containing only contemporaneous and first order connections were insufficient for capturing all sequential dependencies about half the time. Failing to adequately account for sequential dependencies provides inaccurate results and may contribute to faulty substantive inferences.

## Author contributions

AB and PM contributed to the conception and design of the study, and to the analysis and interpretation of the data. AB drafted the manuscript and contributed to the acquisition of the data. PM critically revised the manuscript for important intellectual content. Both authors approved the final version of the manuscript and agreed to be accountable for all aspects of the work. This study was conducted in accordance with and with the approval of the Institutional Review Boards at the universities at which the data were collected. All participants provided written informed consent in accordance with the Declaration of Helsinki.

### Conflict of interest statement

The authors declare that the research was conducted in the absence of any commercial or financial relationships that could be construed as a potential conflict of interest.

## Appendix

**Table:**

white noise test for simulated data da no=199 ni=12 ma=cm cm sy fi=covres.doc mo ny=12 ne=12 ly=id te=ze ps=sy,fi le roi1t1 roi2t1 roi3t1 roi1t2 roi2t2 roi3t2 roi1t3 roi2t3 roi3t3 roi1t4 roi2t4 roi3t4 /	There are 199 observations (no = 199) because 1 degree of freedom was used to estimate the residuals.	There are 12 variables (ni = ny = ne = 12): 3 ROIs at each of 4 time points (contemporaneous, lag 1, lag 2, lag 3).
fr ps(1,1) fr ps(2,1) ps(2,2) fr ps(3,1) ps(3,2) ps(3,3) eq ps(1,1) ps(4,4) ps(7,7) ps(10,10) eq ps(2,1) ps(5,4) ps(8,7) ps(11,10) eq ps(2,2) ps(5,5) ps(8,8) ps(11,11) eq ps(3,1) ps(6,4) ps(9,7) ps(12,10) eq ps(3,2) ps(6,5) ps(9,8) ps(12,11) eq ps(3,3) ps(6,6) ps(9,9) ps(12,12) ou	All variances and covariances of the innovations are freely estimated for the first time point, and then corresponding parameters are set to be equal across lags.	

## References

[B1] Akaike H. (1974). A new look at the statistical model identification. IEEE Trans. Autom. Control 19, 716–723. 10.1109/tac.1974.1100705

[B2] Alonso-Montes C. Diez I. Remaki L. Escudero I. Mateos B. Rosseel Y.. (2015). Lagged and instantaneous dynamical influences related to brain structural connectivity. Front. Psychol. 6:1024. 10.3389/fpsyg.2015.01024 26257682 PMC4508482

[B3] Arbabshirani M. R. Damaraju E. Phlypo R. Plis S. Allen E. Ma S.. (2014). Impact of autocorrelation on functional connectivity. Neuroimage 102, 294–308. 10.1016/j.neuroimage.2014.07.045 25072392 PMC4253536

[B4] Beltz A. M. Gates K. M. Engels A. S. Molenaar P. C. M. Pulido C. Turrisi R.. (2013). Changes in alcohol-related brain networks across the first year of college: a prospective pilot study using fMRI effective connectivity mapping. Addict. Behav. 38, 2052–2059. 10.1016/j.addbeh.2012.12.023 23395930 PMC3616613

[B5] Biswal B. B. Mennes M. Zuo X. N. Gohel S. Kelly C. Smith S. M.. (2010). Toward discovery science of human brain function. Proc. Natl. Acad. Sci. U.S.A. 107, 4734–4739. 10.1073/pnas.0911855107 20176931 PMC2842060

[B6] Boker S. Neale M. Maes H. Wilde M. Spiegel M. Brick T.. (2011). OpenMx: an open source extended structural equation modeling framework. Psychometrika 76, 306–317. 10.1007/s11336-010-9200-6 23258944 PMC3525063

[B7] Box G. E. P. Jenkins G. M. (1970). Time Series Analysis: Forecasting and Control. San Francisco, CA: Holden-Day.

[B8] Brown T. A. (2006). Confirmatory Factor Analysis for Applied Research. New York, NY: GuilfordPress.

[B9] Chang C. Glover G. H. (2009). Effects of model-based physiological noise correction on default mode network anti-correlations and correlations. Neuroimage 47, 1448–1459. 10.1016/j.neuroimage.2009.05.012 19446646 PMC2995588

[B10] Chen G. Glen D. R. Saad Z. S. Hamilton J. P. Thomason M. E. Gotlib I. H.. (2011). Vector autoregression, structural equation modeling, and their synthesis in neuroimaging data analysis. Comput. Biol. Med., 41, 1142–1155. 10.1016/j.compbiomed.2011.09.004 21975109 PMC3223325

[B11] Chou C. P. Bentler P. M. (1990). Model modification in covariance structure modeling: a comparison among likelihood ratio, Lagrange Multiplier, and Wald tests. Multivariate Behav. Res. 25, 115–136. 10.1207/s15327906mbr2501_13 26741976

[B12] Christova P. Lewis S. M. Jerde T. A. Lynch J. K. Georgopoulos A. P. (2011). True associations between resting fMRI time series based on innovations. J. Neural Eng. 8:046025. 10.1088/1741-2560/8/4/046025 21712571

[B13] Cox R. W. (1996). AFNI: software for analysis and visualization of functional magnetic resonance neuroimages. Comput. Biomed. Res. 29, 162–173. 10.1006/cbmr.1996.0014 8812068

[B14] Fox M. D. Zhang D. Snyder A. Z. Raichle M. E. (2009). The global signal and observed anticorrelated resting state brain networks. J. Neurophysiol. 101, 3270–3283. 10.1152/jn.90777.2008 19339462 PMC2694109

[B15] Friston K. J. Josephs O. Zarahn E. Holmes A. P. Rouquette S. Poline J. B. (2000). To smooth or not to smooth? Bias and efficiency in fMRI time-series analysis. Neuroimage 12, 196–208. 10.1006/nimg.2000.0609 10913325

[B16] Friston K. Moran R. Seth A. K. (2013). Analysing connectivity with Granger causality and dynamic causal modelling. Curr. Opin. Neurol. 23, 172–178. 10.1016/j.conb.2012.11.010 23265964 PMC3925802

[B17] Gates K. M. Molenaar P. C. M. (2012). Group search algorithm recovers effective connectivity maps for individuals in homogeneous and heterogeneous samples. Neuroimage 63, 310–319. 10.1016/j.neuroimage.2012.06.026 22732562

[B18] Gates K. M. Molenaar P. C. M. Hillary F. G. Ram N. Rovine M. J. (2010). Automatic search for fMRI connectivity mapping: an alternative to Granger causality testing using formal equivalences among SEM path modeling, VAR, and unified SEM. Neuroimage 50, 1118–1125. 10.1016/j.neuroimage.2009.12.117 20060050

[B19] Gates K. M. Molenaar P. C. M. Hillary F. G. Slobounov S. (2011). Extended unified SEM approach for modeling event-related fMRI data. Neuroimage 54, 1151–1158. 10.1016/j.neuroimage.2010.08.051 20804852

[B20] Granger C. W. J. Newbold P. (1974). Spurious regressions in econometrics. J. Econom. 2, 111–120. 10.1016/0304-4076(74)90034-723794615

[B21] Grant A. M. Fang S.-Y. Li P. (2015). Second language lexical development and cognitive control: a longitudinal fMRI study. Brain Lang. 144, 35–47. 10.1016/j.bandl.2015.03.010 25899988

[B22] Handwerker D. A. Ollinger J. M. D'Esposito M. (2004). Variation of BOLD hemodynamic responses across subjects and brain regions and their effects on statistical analyses. Neuroimage 21, 1639–1651. 10.1016/j.neuroimage.2003.11.029 15050587

[B23] Hillary F. G. Medaglia J. D. Gates K. M. Molenaar P. C. Good D. C. (2014). Examining network dynamics after traumatic brain injury using the extended unified SEM approach. Brain Imaging Behav. 8, 435–445. 10.1007/s11682-012-9205-0 23138853

[B24] Hillary F. G. Medaglia J. D. Gates K. Molenaar P. C. Slocomb J. Peechatka A.. (2011). Examining working memory task acquisition in a disrupted neural network. Brain 134, 1555–1570. 10.1093/brain/awr043 21571783

[B25] Jenkinson M. Beckmann C. F. Behrens T. E. Woolrich M. W. Smith S. M. (2012). FSL. Neuroimage 62, 782–790. 10.1016/j.neuroimage.2011.09.015 21979382

[B26] Jöreskog K. G. Sörbom D. (1992). LISREL. Chicago, IL: Scientific Software International, Inc.

[B27] Kaneoke Y. Donishi T. Iwatani J. Ukai S. Shinosaki K. Terada M. (2012). Variance and autocorrelation of the spontaneous slow brain activity. PLoS ONE 7:e38131 10.1371/journal.pone.0038131 22666461 PMC3364220

[B28] Karunanayaka P. Eslinger P. J. Wang J. L. Weitekamp C. W. Molitoris S. Gates K. M.. (2014). Networks involved in olfaction and their dynamics using independent component analysis and unified structural equation modeling. Hum. Brain Mapp. 35, 2055–2072. 10.1002/hbm.22312 23818133 PMC4557612

[B29] Kim J. Zhu W. Chang L. Bentler P. M. Ernst T. (2007). Unified structural equation modeling approach for the analysis of multisubject, multivariate functional MRI data. Hum. Brain Mapp. 28, 85–93. 10.1002/hbm.20259 16718669 PMC6871502

[B30] Loehlin J. C. (1996). The Cholesky approach: a cautionary note. Behav. Genet. 26, 65–69. 10.1007/BF02361160

[B31] Lütkepohl H. (2005). New Introduction to Multiple Time Series Analysis. Berlin: Springer.

[B32] Mathworks (2013). Matlab. Natick, MA: The Mathworks, Inc.

[B33] McIntosh A. R. Gonzalez-Lima F. (1994). Structural equation modeling and its application to network analysis in functional brain imaging. Hum. Brain Mapp. 2, 2–22. 10.1002/hbm.46002010419501173

[B34] Molenaar P. C. M. (1985). A dynamic factor model for the analysis of multivariate time series. Psychometrika 50, 181–202. 10.1007/BF02294246

[B35] Molenaar P. C. M. Lo L. L. (2015). Alternative forms of granger causality, heterogeneity and non-stationarity, in Statistics and Causality, eds von Eye A. Wiedermann W. (Hoboken, NJ: Wiley).

[B36] Murphy K. Birn R. M. Bandettini P. A. (2013). Resting-state fMRI confounds and cleanup. Neuroimage 80, 349–359. 10.1016/j.neuroimage.2013.04.001 23571418 PMC3720818

[B37] Murphy K. Birn R. M. Handwerker D. A. Jones T. B. Bandettini P. A. (2009). The impact of global signal regression on resting state correlations: are anti-correlated networks introduced? Neuroimage 44, 893–905. 10.1016/j.neuroimage.2008.09.036 18976716 PMC2750906

[B38] Nichols T. T. Gates K. M. Molenaar P. C. M. Wilson S. J. (2014). Greater BOLD activity but more efficient connectivity is associated with better cognitive performance within a sample of nicotine-deprived smokers. Addict. Biol. 19, 931–940. 10.1111/adb.12060 23573872 PMC3711955

[B39] Pashler H. Wagenmakers E.-J. (2012). Editors' introduction to the special section on replicability in psychological science: a crisis of confidence? Perspect. Psychol. Sci. 7, 528–530. 10.1177/1745691612465253 26168108

[B40] Penny W. Harrison L. (2007). Multivariate autoregressive models, in Statistical Parametric Mapping: The Analysis of Functional Brain Images, eds Friston K. J. Ashburner J. T. Kiebel S. J. Nichols T. E. Penny W. D. (London: Elsevier), 534-540.

[B41] Penny W. Kiebel S. Friston K. J. (2003). Variational Bayesian inference for fMRI time series. Neuroimage 19, 727–741. 10.1016/s1053-8119(03)00071-5 12880802

[B42] Poldrack R. A. (2000). Imaging brain plasticity: conceptual and methodological issues - A theoretical review. Neuroimage 12, 1–13. 10.1006/nimg.2000.0596 10875897

[B43] Raichle M. E. MacLeod A. M. Snyder A. Z. Powers W. J. Gusnard D. A. Shulman G. L. (2001). A default mode of brain function. Proc. Natl. Acad. Sci. U.S.A. 98, 676–682. 10.1073/pnas.98.2.676 11209064 PMC14647

[B44] Ramsey J. D. Hanson S. J. Hanson C. Halchenko Y. O. Poldrack R. A. Glymour C. (2010). Six problems for causal inference from fMRI. Neuroimage 49, 1545–1558. 10.1016/j.neuroimage.2009.08.065 19747552

[B45] Rosseel Y. (2012). lavaan: an R package for structural equation modeling. J. Stat. Softw. 48, 1–36. 25601849

[B46] Smith S. M. (2012). The future of FMRI connectivity. Neuroimage 62, 1257–1266. 10.1016/j.neuroimage.2012.01.022 22248579

[B47] Smith S. M. Miller K. L. Salimi-Khorshidi G. Webster M. Beckmann C. F. Nichols T. E.. (2011). Network modelling methods for fMRI. Neuroimage 54, 875–891. 10.1016/j.neuroimage.2010.08.063 20817103

[B48] Sörbom D. (1989). Model modification. Psychometrika 54, 371–384. 10.1007/bf022946235504613

[B49] Turrisi R. Mallett K. A. Cleveland M. J. Varvil-Weld L. Abar C. Scaglione N.. (2013). Evaluation of timing and dosage of a parent-based intervention to minimize college students' alcohol consumption. J. Stud. Alcohol Drugs 74, 30–40. 10.15288/jsad.2013.74.30 23200148 PMC3517262

[B50] Van Dijk K. R. A. Hedden T. Venkataraman A. Evans K. C. Lazar S. W. Buckner R. L. (2010). Intrinsic functional connectivity as a tool for human connectomics: theory, properties, and optimization. J. Neurophysiol. 103, 297–321. 10.1152/jn.00783.2009 19889849 PMC2807224

[B51] Varvil-Weld L. Mallett K. A. Turrisi R. Abar C. C. (2012). Using parental profiles to predict membership in a subset of college students experiencing excessive alcohol consequences: findings from a longitudinal study. J. Stud. Alcohol Drugs 73, 434–443. 10.15288/jsad.2012.73.434 22456248 PMC3316715

[B52] Varvil-Weld L. Scaglione N. Cleveland M. J. Mallett K. A. Turrisi R. Abar C. C. (2014). Optimizing timing and dosage: does parent type moderate the effects of variations of a parent-based intervention to reduce college student drinking? Prev. Sci. 15, 94–102. 10.1007/s11121-012-0356-4 23404668 PMC3688671

[B53] Wilson S. J. Sayette M. A. Fiez J. A. (2012). Quitting-unmotivated and quitting-motivated cigarette smokers exhibit different patterns of cue-elicited brain activation when anticipating an opportunity to smoke. J. Abnorm. Psychol. 121, 198–211. 10.1037/a0025112 21859165 PMC3704179

[B54] Woolrich M. W. Ripley B. D. Brady M. Smith S. M. (2001). Temporal autocorrelation in univariate linear modeling of fMRI data. Neuroimage 14, 1370–1386. 10.1006/nimg.2001.0931 11707093

[B55] Wu G. R. Liao W. Stramaglia S. Ding J. R. Chen H. Marinazzo D. (2013). A blind deconvolution approach to recover effective connectivity brain networks from resting state fMRI data. Med. Image Anal. 17, 365–374. 10.1016/j.media.2013.01.003 23422254

[B56] Yang J. Gates K. M. Molenaar P. Li P. (2015). Neural changes underlying successful second language word learning: an fMRI study. J. Neurolinguistics 33, 29–49. 10.1016/j.jneuroling.2014.09.004

